# Excessive Daytime Sleepiness and Injury of the Ascending Reticular Activating System Following Whiplash Injury

**DOI:** 10.3389/fnins.2018.00348

**Published:** 2018-05-29

**Authors:** Sung H. Jang, Seong H. Kim, Young H. Kwon

**Affiliations:** ^1^Department of Physical Medicine and Rehabilitation, College of Medicine, Yeungnam University, Daegu, South Korea; ^2^Department of Neurosurgery, College of Medicine, Yeungnam University, Daegu, South Korea

**Keywords:** excessive daytime sleepiness, ascending reticular activating system, hypersomnia, whiplash injury, diffusion tensor tractography

## Abstract

**Objectives:** This study investigated injuries of the ascending reticular activating system (ARAS) following whiplash injury, in patients with excessive daytime sleepiness (EDS).

**Methods:** Twenty-three patients with whiplash injury and 26 healthy control subjects were recruited for this study. Epworth Sleepiness Scale (ESS) was used for evaluating sleepiness. According to the ESS score, the patients were classified into two groups: subgroup A – ESS score <10, and subgroup B – ESS score ≥10. Three components of the ARAS (lower dorsal, lower ventral, and upper) were evaluated for fractional anisotropy (FA) and tract volume (TV).

**Results:** No significant differences were observed in the FA and TV values of the lower dorsal and upper ARAS between the patient and control groups (*p* > 0.05). Conversely, the values of FA and TV in the lower ventral ARAS of the patient group were significantly lower than those of the control group (*p* < 0.05). Comparing the values of subgroups A and B, the TV value of subgroup B was significantly lower than subgroup A (*p* < 0.05). However, no significant differences were observed in the values of the FA and TV in the lower dorsal and upper ARAS, and the FA value in the lower ventral ARAS (*p* > 0.05).

**Conclusions:** We found significant injury of the lower ventral ARAS in EDS patients with whiplash injury. These results suggest that diffusion tensor tractography (DTT) could provide useful information for detecting injuries of the ARAS following whiplash injury, in patients with EDS.

## Introduction

Whiplash is a bony or soft tissue injury resulting from an acceleration-deceleration mechanism of energy transfer to the neck (Spitzer et al., 1995). Various symptoms suggesting brain injury, including sleeping problems, cognitive impairment, visual impairment, and vestibular symptoms, have been reported following whiplash injury (Ettlin et al., 1992). Previous studies on functional neuroimaging, brain MRI and dissection of post-mortem brain have also suggested evidences of brain injury following whiplash injury (Otte et al., 1997; Obermann et al., 2009; Vállez García et al., 2016). Since the introduction of diffusion tensor imaging (DTI), is a technique that allows

for evaluation of the integrity of white matter tracts by virtue of its ability to image water diffusion characteristics, several studies using diffusion tensor tractography (DTT), which is reconstructed from DTI data, have reported axonal injuries of several neural tracts of the brain including the ascending reticular activating system (ARAS) following whiplash injury (Mori et al., 1999; Kwon and Jang, 2014; Jang and Kwon, 2015a, 2017; Seo and Jang, 2015; Jang et al., 2016b,d; Jang and Lee, 2017). However, the brain injury following whiplash injury has so far not been clearly elucidated.

DTT allows three-dimensional reconstruction and estimation of majority of the ARAS the human brain including the lower dorsal ARAS between the pontine reticular formation and the intralaminar thalamic nuclei which is mainly involved in alertness, the lower ventral ARAS between the pontine RF and the hypothalamus which is closely related with sleep, and the upper ARAS between the intralaminar nuclei of the thalamus and the cerebral cortex which is involved in awareness (Paus, 2000; Zeman, 2001; Yeo et al., 2013; Jang et al., 2014, 2016b; Jang and Kwon, 2015b; Jang and Lee, 2017).

The prevalence of sleeping problems following whiplash injury is high (34–62%), but little is known about excessive daytime sleepiness (EDS) following whiplash injury. By contrast, EDS is a common sequela with a high prevalence (14–57%) in traumatic brain injury (TBI) patients (Castriotta et al., 2007; Watson et al., 2007; Castriotta and Murthy, 2011; Ouellet et al., 2015). Injury of several neural structures including hypothalamus, ascending reticular activating system (ARAS), cerebral cortex, and brainstem has been suggested to be related with EDS following TBI (Baumann et al., 2005, 2009; Valko et al., 2015; Jang and Kwon, 2016; Jang et al., 2016c). On the other hand, a few case studies using diffusion tensor tractography (DTT), which is reconstructed from diffusion tensor imaging (DTI), DTI data DTT demonstrated that injury of the ARAS, especially the lower ventral ARAS, is related with EDS following whiplash injury (Jang et al., 2016b; Jang and Kwon, 2017). However, the relation between EDS and injury of the ARAS following whiplash is yet not elucidated.

In the current study, we investigated the ARAS injury in patients afflicted with EDS following whiplash injury, using DTT.

## Methods

### Subjects

A total of 23 consecutive patients with whiplash injury (September 2014 to December 2017) (8 males, 15 females; mean age 44.7 years, range 26–61 years) and 26 age- and sex-matched normal control subjects (12 males, 14 females; mean age 41.5 years, range 27–62 years) with no history of neurologic or psychiatric disease were recruited into this study. Inclusion criteria for the 23 patients were as follows: (1) more than 2 weeks after the onset of whiplash injury, (2) flexion-hyperextension injury of the head and neck following motor vehicle collision, (3) loss of consciousness for ≤30 min, post-traumatic amnesia for ≤24 h, and initial Glasgow Coma Scale score of 13–15, (4) no specific lesion observed on brain MRI (T1-weighted, T2-weighted, and fluid attenuated inversion recovery images), (5) age at the time of head trauma over 18 years, and (6) no definite cognitive impairment which was evaluated by Mini-Mental State Exam (full score: 30, cut-off score: 25) and (7) no previous history of head trauma, neurologic or psychiatric disease (Folstein et al., 1975). This was a retrospective study, and this study was carried out in accordance with the recommendations of “The CARE of guidelines” with written informed consent from all subjects. The patient signed a written informed consent in accordance with the Declaration of Helsinki, and the study protocol was approved by the Institutional Review Board of the Yeungnam University hospital.

### Clinical evaluation

The Epworth Sleepiness Scale (ESS, cut-off value: 10 scores, full mark: 24 scores, higher score means more sleepiness) was used for evaluation of sleepiness at the time of DTI scanning (Johns, 1992). We classified the 23 patients into two subgroups according to the ESS cut-off score: 9 non-EDS patients belonged to subgroup A (ESS < 10), and 14 EDS patients were assigned to subgroup B (ESS ≥ 10). No significant difference was observed in age and sex between the patient and control groups, and between subgroups A and B (*p* > 0.05) (Table 1). However, the ESS score was significantly higher in subgroup B than in subgroup A (*p* > 0.05).

**Table 1:** Demographic and clinical data of the patient and control groups.

	**Patient group (*****n*** = **23)**			**Control group (*n* = 26)**
	**Subgroup A (*n* = 9)**	**Subgroup B (*n* = 14)**	**Total**	
Sex (male: female)	3:6	5:9	8:15	12:14
Mean age, years	47.2 (±12.4)	43.1 (±9.3)	44.7 (±10.6)	41.5 (±9.6)
ESS	7.1 (±2.2)*	15.3 (±4.3)*	12.1 (±5.4)	–
LOC, minutes	2.5 (±1.0)	4.7(±5.0)	3.7 (±4.9)	–
PTA, minutes	7.1 (±11.3)	28.3 (±54.1)	21.9 (±43.8)	–
GCS score	15.0 (±0.0)	15.0 (±0.0)	15.0 (±0.0)	–
Mean duration to DTI (months)	3.9 (±5.2)	7.5 (±7.3)	6.5 (±6.7)	–

*Values represent mean (±standard deviation); ESS, Epworth Sleepiness Scale; LOC, loss of consciousness; PTA, post-traumatic amnesia; GCS, Glasgow Coma Scale; DTI, diffusion tensor imaging*.

* *significantly different of Epworth Sleepiness Scale between subgroups A and B at p < 0.05*.

### Diffusion tensor imaging and fiber tracking

Acquisition of DTI data was performed 6.5 ± 6.7 months after onset of the whiplash injury, using a 6-channel head coil on a 1.5-T Philips Gyroscan Intera (Philips, Best, The Netherlands) and single-shot echo-planar imaging. For each of the 32 noncollinear diffusion sensitizing gradients, 67 contiguous slices were acquired parallel to the anterior commissure-posterior commissure line. Fiber tracking was performed using a probabilistic tractography method based on a multifiber model using tractography routines implemented in FMRIB Diffusion. Three portions of the ascending reticular activating system (ARAS) were reconstructed by selection of fibers passing through the following regions of interest (ROIs): the lower dorsal ARAS (seed ROI: the pontine RF, and target ROI: the ILN), the lower ventral ARAS (seed ROI: the pontine RF, and target ROI: the hypothalamus), and the upper ARAS (the neural connectivity of the ILN to the cerebral cortex) (Jang et al., 2016b; Jang and Kwon, 2017). Of the 5,000 samples generated from the seed voxel, results for contact were visualized at a minimum threshold for the lower (dorsal and ventral) ARAS of 2, and for neural connectivity of the ILN of 10 streamlined through each voxel for analysis. Values of fractional anisotropy (FA) and tract volume (TV) of the three ARAS regions were measured.

### Statistical analysis

Statistical analysis was performed using the SPSS 12.0 for Windows (SPSS Inc., Chicago, IL, USA). The group differences in distribution of age and EES were assessed by independent *t*-tests. The Chi-Square Test was used to verify gender differences between the patient and control groups, and between subgroups A and B. The independent *t*-test was used for determining differences in the values of FA and TV between the patient and control groups, and differences between subgroups A and B in terms of DTT parameters (FA and TV). The significant level of the *p*-value was set at 0.05.

## Results

Comparison of the DTT parameters of lower dorsal, ventral and upper ARAS between the patient and control groups is summarized in Table 2. No significant differences were observed in the FA and TV values of the lower dorsal and upper ARAS between the patient and control groups (*p* > 0.05). By contrast, the values of FA and TV in the lower ventral ARAS of the patient group were significantly different than the control group (*p* < 0.05).

**Table 2:** Comparison of diffusion tensor imaging parameters of the patient and control groups.

	**Lower dorsal ARAS**		**Lower ventral ARAS**		**Upper ARAS**	
	**FA**	**TV**	**FA**	**TV**	**FA**	**TV**
Patient group	0.40 (±0.04)	350.26 (±168.93)	0.36 (±0.04)	189.34 (±138.11)	0.35 (±0.03)	20354.54 (±5958.37)
Control group	0.41 (±0.03)	375.58 (±152.75)	0.39 (±0.42)	323.23 (±120.80)	0.36 (±0.01)	19340.92 (±5559.88)
*p*-value	0.127	0.441	0.001*	0.001*	0.104	0.388

*Values represent mean (±standard deviation); ARAS, ascending reticular activating system; FA, fractional anisotropy; TV, tract volume*.

* *significant differences between the patient and control groups at p < 0.05*.

Comparing subgroups A and B, the TV value of lower ventral ARAS was significantly different between the subgroups (*p* < 0.05). However, no significant difference was observed in the values of the FA and TV in the lower dorsal and upper ARAS, and FA value in the lower ventral ARAS (*p* > 0.05) (Table 3).

**Table 3:** Comparison of diffusion tensor imaging parameters of the subgroups A and B.

	**Lower dorsal ARAS**		**Lower ventral ARAS**		**Upper ARAS**	
	**FA**	**TV**	**FA**	**TV**	**FA**	**TV**
Subgroup A	0.41 (±0.05)	330 (±165.70)	0.36 (±0.04)	262 (±147.55)	0.35 (±0.02)	19725.61 (±3852.02)
Subgroup B	0.39 (±0.04)	363.29 (±172.64)	0.36 (±0.04)	132.48 (±100.79)	0.35 (±0.03)	20758.85 (±7028.13)
*p*-value	0.127	0.441	0.836	0.003*	0.899	0.524

*Values represent mean (±standard deviation); ARAS, ascending reticular activating system; FA, fractional anisotropy; TV, tract volume*.

* *significant differences between the subgroup A and B at p < 0.05*.

## Discussion

**Figure 1:**
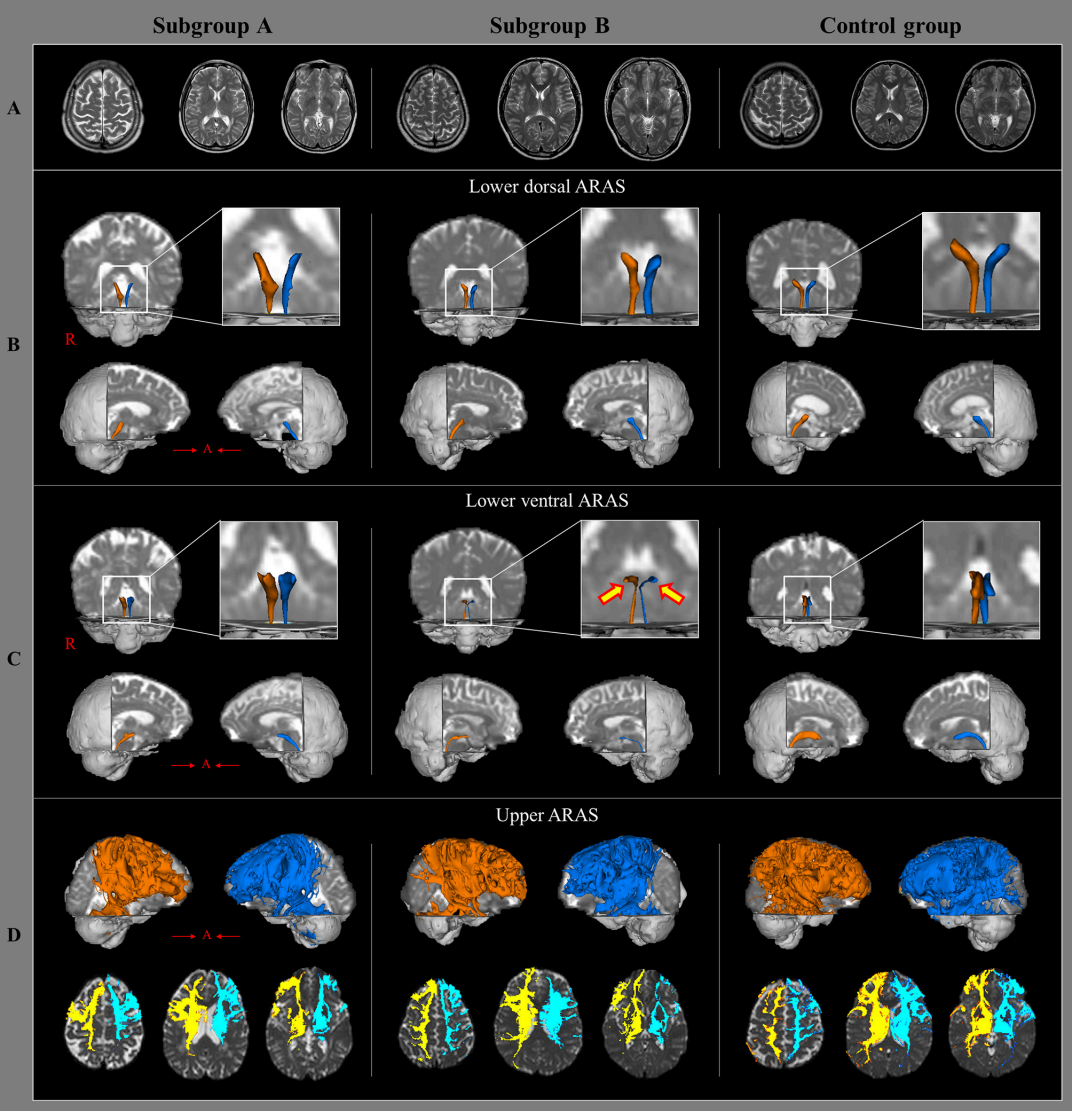
Brain MR images and diffusion tensor tractography of the three components of ascending reticular activating system, in a representative patient and control subject. **(A)** T2-weighted brain MR images at the time of diffusion tensor imaging scanning in a patient with subgroup A (50-year old male) and subgroup B(35-year old male), and a control subject (63-year old female) show no abnormality. **(B)** Results of diffusion tensor tractography (DTT) for the lower dorsal ascending reticular activating system (ARAS). **(C)** Results of DTT for the lower ventral ARAS: the lower ventral ARAS in the patient with subgroup B is narrowed in the patient on both sides (arrows), compared with those of the control subject. **(D)** Results of DTT for the upper ARAS.

In this study, injury of the ARAS was investigated in patients with whiplash injury and we found the results; the value of TV in the lower ventral ARAS in subgroup A was lower than the subgroup B. Among the DTT parameters, FA and TV values are most commonly used in evaluating the state of neural tracts in patients with brain injury (Mori et al., 1999; Jang et al., 2016b). The FA value represents the white matter organization by indicating the degree of directionality and integrity of white matter microstructures such as axon, myelin, and microtubule (Mori et al., 1999). The TV value indicates the included number of voxels in a neural tract. Therefore, decrement of FA or TV is indicative of a neural tract injury. Our results indicate injury to the lower ventral ARAS following an episode of whiplash, especially in patients with EDS (Figure 1). We believe that the lower ventral ARAS is more vulnerable to the acceleration-deceleration strain during a whiplash injury since it is located close to the neck, and has an anatomical characteristic with an anteriorly oblique feature as compared to the lower dorsal ARAS (Spitzer et al., 1995; Jang et al., 2016b; Jang and Kwon, 2017).

The hypothalamus, which is an endpoint of the ventral ARAS, is regarded as a key regulator of sleep and wakefulness. Many studies report an association between injury of the hypothalamus with sleep disorders, such as narcolepsy and hypersomnia, following TBI (Baumann et al., 2005, 2009; Valko et al., 2015; Jang and Kwon, 2016; Jang et al., 2016b,c; Jang and Lee, 2017). In addition, since the reconstruction of the ARAS using DTT is possible, many studies have demonstrated that the lower ventral ARAS, which is connected to the hypothalamus, is closely related with hypersomnia following brain injury (Jang and Kwon, 2016, 2017; Jang et al., 2016a,b, 2017, in press). Therefore, the results of our study appear to be consistent with previous studies (Jang and Kwon, 2016, 2017; Jang et al., 2016a,b, 2017, in press).

After introduction of DTI, several studies have reported EDS in patients with TBI (Jang and Kwon, 2016, 2017; Jang et al., 2016c; Puligheddu et al., 2017). In 2016, Jang and Kwon reported two patients who presented with fatigue and EDS, showed injuries of the lower dorsal and ventral ARAS following mild TBI (Jang and Kwon, 2016). During the same year, Jang et al. investigated the relationship between EDS and injury of the hypothalamus in 53 patients with mild TBI (Jang et al., 2016c). They found injury of the hypothalamus in 32 patients with EDS, which correlated with injury severity of the hypothalamus (Jang et al., 2016c). Recently, Puligheddu et al reported a patient with EDS after a severe TBI, revealed injury of the frontal white matter which appeared to be a part of the upper ARAS (Puligheddu et al., 2017). Regarding EDS related with whiplash injury, two case studies with mild TBI are reported (Jang et al., 2016b; Jang and Kwon, 2017). In 2015, Jang and Kwon reported a patient with mild TBI who showed narcolepsy, including EDS and injury of the lower ventral ARAS following whiplash injury (Jang et al., 2016b). In 2017, a patient revealed aggravation of EDS (ESS: 12 scores at 10 weeks after onset, and 18 scores at 16 months after onset) with progression of the injury of the lower dorsal and ventral (mainly) ARAS on follow up DTTs (Jang and Lee, 2017). Thus, to the best of our knowledge, our study is the first to demonstrate the association between EDS and injury of the ARAS in a large number of subjects with whiplash injury.

However, limitations of this study need to be considered. First, the DTT analysis is operator-dependent, and regions of fiber complexity and crossing can prevent full reflection of the underlying fiber architecture (Yamada et al., 2009). Second, since this study was performed retrospectively, we were not able to measure the degree of EDS using an objective measure such as Multiple Sleep Latency Test (Castriotta and Murthy, 2011). Therefore, further prospective studies that include the objective measures should be encouraged. Third, since patients were recruited from amongst those who visited the rehabilitation department of a university hospital, there was a possibility that patients with severe clinical manifestations might be included in this study, as compared to all patients with whiplash injury.

In conclusion, we found the injury of the lower ventral ARAS in patients with whiplash injury, especially in patients with EDS. These results suggest that DTT could provide useful information in detecting injuries of the ARAS in patients with EDS following whiplash injury, which otherwise remain undetected on conventional brain MRI in patients with mild TBI.

## Author contributions

SJ: study concept and design, acquisition of data; SK: acquisition of data, interpretation of data; YK: critical revision of manuscript for intellectual content.

### Conflict of interest statement

The authors declare that the research was conducted in the absence of any commercial or financial relationships that could be construed as a potential conflict of interest.
